# Myeloid cells in circulation and tumor microenvironment of breast cancer patients

**DOI:** 10.1007/s00262-017-1977-z

**Published:** 2017-03-10

**Authors:** Salman M. Toor, Azharuddin Sajid Syed Khaja, Haytham El Salhat, Issam Faour, Jihad Kanbar, Asif A. Quadri, Mohamed Albashir, Eyad Elkord

**Affiliations:** 1 College of Medicine and Health Sciences, United Arab Emirates University, Al Ain, United Arab Emirates; 2 Cancer Research Center, Qatar Biomedical Research Institute, College of Science and Engineering, Hamad Bin Khalifa University, Qatar Foundation, Doha, Qatar; 3 Surgery Department, Tawam Hospital, Al Ain, United Arab Emirates; 4 Oncology Department, Al Noor Hospital, Abu Dhabi, United Arab Emirates; 5 Oncology Department, Tawam Hospital, Al Ain, United Arab Emirates; 6 Pathology Department, Tawam Hospital, Al Ain, United Arab Emirates; 7 Institute of Cancer Sciences, University of Manchester, Manchester, UK

**Keywords:** Myeloid cells, Myeloid-derived suppressor cells, Neutrophils, Breast cancer, Tumor microenvironment, Circulation

## Abstract

Pathological conditions including cancers lead to accumulation of a morphological mixture of highly immunosuppressive cells termed as myeloid-derived suppressor cells (MDSC). The lack of conclusive markers to identify human MDSC, due to their heterogeneous nature and close phenotypical and functional proximity with other cell subsets, made it challenging to identify these cells. Nevertheless, expansion of MDSC has been reported in periphery and tumor microenvironment of various cancers. The majority of studies on breast cancers were performed on murine models and hence limited literature is available on the relation of MDSC accumulation with clinical settings in breast cancer patients. The aim of this study was to investigate levels and phenotypes of myeloid cells in peripheral blood (*n* = 23) and tumor microenvironment of primary breast cancer patients (*n* = 7), compared with blood from healthy donors (*n* = 21) and paired non-tumor normal breast tissues from the same patients (*n* = 7). Using multicolor flow cytometric assays, we found that breast cancer patients had significantly higher levels of tumor-infiltrating myeloid cells, which comprised of granulocytes (*P* = 0.022) and immature cells that lack the expression of markers for fully differentiated monocytes or granulocytes (*P* = 0.016). Importantly, this expansion was not reflected in the peripheral blood. The immunosuppressive potential of these cells was confirmed by expression of Arginase 1 (ARG1), which is pivotal for T-cell suppression. These findings are important for developing therapeutic modalities to target mechanisms employed by immunosuppressive cells that generate an immune-permissive environment for the progression of cancer.

**Electronic supplementary material:**

The online version of this article (doi:10.1007/s00262-017-1977-z) contains supplementary material, which is available to authorized users.

## Introduction

Breast cancer is the most commonly diagnosed cancer and the leading cause of cancer-related deaths in females worldwide [1]. The striking mortality rates are caused by metastasis to distant regions from the primary tumor, while recent advances in improving survival rates are attributed to early detection through screening and initiation of neoadjuvant therapy in patients [2]. The developments of novel approaches to identify prognostic markers for patients who are at high risk of developing breast cancer are, therefore, of cardinal significance.

Human carcinomas induce an immune response in the tumor microenvironment (TME) [3]. However, emerging evidence has established the role of different immunosuppressive cells, such as myeloid-derived suppressor cells (MDSC) and regulatory T cells (Treg), in cancer bearing hosts. MDSC are a heterogeneous population of myeloid progenitor and activated myeloid cells, halted at varying stages of maturation and differentiation that exhibit a potent immunosuppressive activity against T-cell responses [4]. Myeloid cells generated in bone marrow differentiate into mature granulocytes, macrophages or dendritic cells. However, pathological conditions such as cancers result in accumulation of a morphological mixture of granulocytic and monocytic MDSC in TME and circulation through various factors produced by tumors or activated T cells [5]. The heterogeneous nature of MDSC together with functional and phenotypical overlap with other myeloid populations has, therefore, made it challenging to identify these cells. The majority of studies on MDSC in breast cancer have been carried out on murine models by developing spontaneous tumors due to predisposing mutations leading to breast cancer development or through transplantation of tumors in xenografts [6]. Limited data are available for the conclusive phenotypical profiling of myeloid cells in circulation and the TME of breast cancer patients and their correlation with clinical settings.

MDSC in humans are commonly defined as cells which express common myeloid markers CD33 and CD11b but lack the expression of HLA-DR, and are further divided into monocytic MDSC (M-MDSC) or granulocytic MDSC (G-MDSC) based on the expression of CD14 and CD15, respectively, while immature MDSC (IM-MDSC) or early-stage MDSC (e-MDSC) lack CD14 and CD15 expression [7]. MDSC exert their suppressive role through increased production of suppressive factors such as Arginase 1 (ARG1), nitric oxide and reactive oxygen and/or reactive nitrogen species along with modulating the production of various cytokines [8]. Several studies have shown the phenotypical and functional similarities between G-MDSC and neutrophils [9]. The term G-MDSC has recently been revised to polymorphonuclear MDSC (PMN-MDSC) to differentiate between steady-state neutrophils and G-MDSC, which have fewer granules and increased ARG1 and CD11b expression [7]. Immunosuppression by tumor-associated neutrophils (TAN) uses similar mechanisms as MDSC and elevated neutrophil to lymphocyte ratio (NLR) is considered as a poor prognostic factor in cancer patients [10].

In this study, we investigated the phenotypes and levels of myeloid cells in circulation and tumors from primary breast cancer (PBC) patients, and compared their levels with peripheral blood from healthy donors and paired, adjacent non-tumor breast tissue, respectively. We found that the immune profile of the TME of breast cancer patients is not reflected in circulation; there was an expansion of granulocytic and immature myeloid cells in the tumors but not in the periphery. Furthermore, there was no association between levels of circulating myeloid cells and patients’ TNM stage or histological grade. This disparity in peripheral blood and tumors provides a better understanding of the role of myeloid cells in the TME of breast cancer patients, and therefore, offers new facets for the development of therapeutic modalities to target the expanded immunosuppressive populations in the TME.

## Materials and methods

### Ethical approval and study subjects

The study was conducted with an ethical approval from Al Ain Medical District Research Ethics committee, Al Ain, United Arab Emirates (Protocol No. 13/23-CRD 244/13). All participating individuals provided written informed consent before sample collection. Peripheral blood from healthy donors (HD, *n* = 21) and primary breast cancer patients (PBC, *n* = 23) were collected in heparinized tubes (200 IU). Tumor and paired, adjacent non-tumor breast tissue specimens were collected from breast cancer patients (*n* = 7) following surgery. Patients did not receive any treatment prior to sample collection. Table 1 shows the clinical and pathological characteristics of all participating individuals.

**Table 1:** Characteristic features of study populations

	HD	PBC
Number	21	23
Age (median)	29 (19–51)^a^	48 (27–65)^a^
Gender (Male: female)	9:12	0:23
TNM stage		
I		9 (3)^b^
II		11 (3)^b^
III		3 (1)^b^
Tumor size (cm)		2.2 (0.8–4.5)^a^
Histological grade		
Well/moderate		11
Poor/undifferentiated		12
Lymph node invasion		9
Estrogen receptor (ER) positive/negative		16/7
Progesterone receptor (PR) positive/negative		13/10
Triple Negative		2
Ki-67 expression		
≤30%		10
>30%		8
No information		5

*HD* healthy donor, *PBC* primary breast cancer

^a^ Data shown represent median (range)

^b^ Samples taken from patients for investigating tissue-infiltrating myeloid cells

### Enzyme disaggregation of tumor and normal tissues for cell isolation

Enzyme disaggregation (ED) of fresh tumor and normal tissues from breast cancer patients, collected in cold RPMI-1640 media was performed on a rollover mixer at 37 °C for 60 min. Briefly, tissues were first washed with phosphate buffered saline (PBS) and then mechanically cut into small fragments (2–4 mm) using a surgical scalpel. Tissues were then suspended into RPMI-1640 media with 1% Penicillin/Streptomycin and an enzyme cocktail, consisting of 1 mg/ml Collagenase (Sigma–Aldrich, Dorset, UK), 100 µg/ml Hyaluronidase type V (Sigma–Aldrich) and 30 IU/ml of Deoxyribonuclease I (Sigma–Aldrich). Cell suspension was then passed through a 100 µm BD Falcon cell strainer (BD Biosciences, Oxford, UK) to remove debris and aggregates. Cells were then resuspended in RPMI-1640 media enriched with 10% FCS and 1% Penicillin/Streptomycin (complete medium) after washing with RPMI-1640 media.

### Surface and intracellular staining of whole blood for flow cytometric analyses

**Fig. 1:**
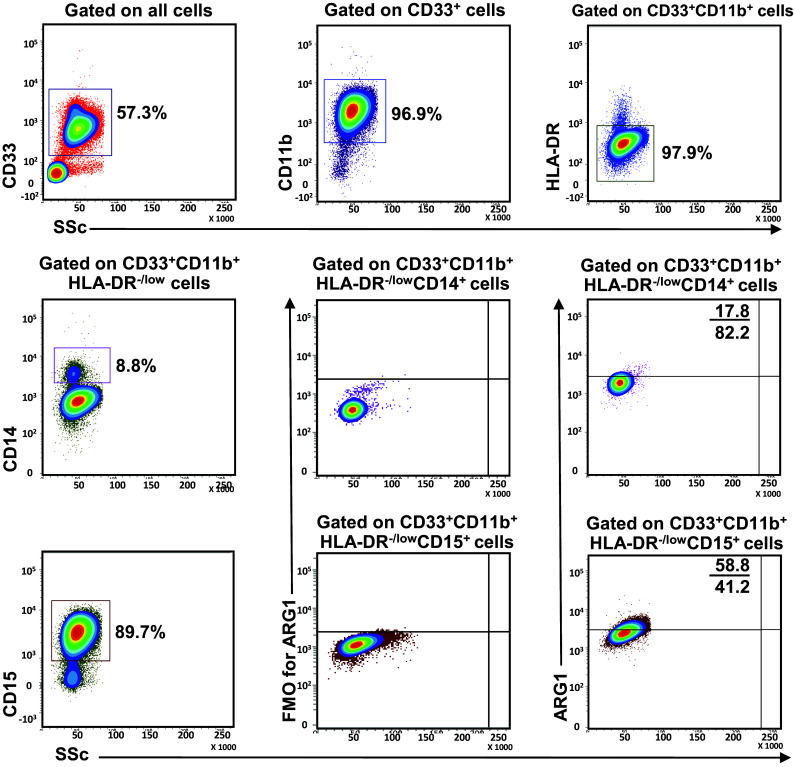
Gating strategy of myeloid cells. Representative flow cytometric plots showing the gating strategy used to identify myeloid cells in peripheral blood of HD and PBC patients. Fresh whole blood from a PBC patient was stained for MDSC markers. CD33^+^ cells were gated first from live cells, followed by gating CD11b^+^ cells within the CD33^+^ parent population and then HLA-DR^−/low^ cells from CD33^+^CD11b^+^ parent population. Monocytic myeloid cells were identified as CD14^+^ cells, while granulocytic myeloid cells were identified based on the expression of CD15. ARG1 expression in each subset was recorded by gating the corresponding parent populations, respectively. FMO controls for ARG1 staining for M-MDSC and N/G-MDSC are shown

Following collection, all blood samples were stained on the same day. 200 µl blood from each sample was used for whole blood staining for MDSC markers; 100 µl used as nonstained control and 100 µl stained for each sample. Mouse anti-human CD33-APC (Clone WM53), mouse anti-human CD11b-APC-Cy7 (Clone ICRF44), mouse anti-human HLA-DR-PE (Clone G46-6), mouse anti-human CD14-PerCP-Cy5.5 (Clone M5E2) and mouse anti-human CD15-PE-Cy7 (Clone HI98) antibodies were added to the stained samples. All antibodies used were purchased from BD Biosciences. Tubes were incubated at 4 °C for 25 min. RBC lysis buffer (BD FACS Lysing solution) was then added to each tube and incubated in the dark for 5 min. After washing samples twice with PBS, cells were fixed and permeabilized using fixation/permeabilization buffer (eBioscience, San Diego, USA), vortexed thoroughly and incubated at 4 °C for 45 min. Samples were then washed twice with permeabilization wash buffer (eBioscience) and stained with sheep anti-human/mouse Arginase 1-FITC antibody (ARG1; R&D Systems, Minneapolis, USA) for intracellular staining and incubated at 4 °C for 25 min, followed by two washes with wash buffer (eBioscience). The cell pellet was resuspended in 300 µl of flow cytometry staining buffer (eBioscience) and analyzed on BD FACSCanto II flow cytometer (BD Biosciences, San Jose, USA). Fluorescence minus one (FMO) controls were used to identify positive populations for ARG1 (Fig. 1) and all other markers (data now shown). However, day to day variations in measurements cannot be fully excluded.

### Staining of tissue-infiltrating immune cells for flow cytometric analyses

Staining of immune cells extracted by ED was performed by blocking the Fc receptor using FcR Blocker (Miltenyi Biotec, Bergisch Gladbach, Germany). 7AAD viability dye (eBioscience) was then added, followed by staining with mouse anti-human CD11b-APC-Cy7 (BD Biosciences), mouse anti-human CD33-FITC (BioLegend, San Diego, USA), mouse anti-human HLA-DR-PE (BD Biosciences), CD14-PE-Cy7 (eBioscience) and mouse anti-human CD15-APC (BioLegend). After incubation at 4 °C for 25 min, samples were washed twice with PBS and the pellets were resuspended in 300 µl flow cytometry staining buffer (eBioscience) and analyzed using BD FACSCanto II flow cytometer. Some tumor-infiltrating immune cells were also stained for ARG1 expression, as described above, with the addition of Fixable Viability Dye eFluor^®^ 780 (FVD780; eBioscience) to gate live cells. Flow cytometric data were analyzed using BD FACSuite software (BD Biosciences).

### Statistical analyses

Statistical analyses were performed using GraphPad Prism 5.0 software (GraphPad Software, San Diego, USA). Shapiro–Wilk normality test followed by paired/Wilcoxon matched-pairs signed rank test or unpaired/Mann–Whitney tests were used to examine the differences within groups or between groups, respectively. A *P* value of ≤0.05 was considered statistically significant. The data are presented as means ± SEM with the levels of cells measured as relative percentages or calculated percentages from parent population(s). The relative percentage of each population subset was multiplied by the relative percentage of its respective parent population and the resulting value was presented as calculated percentage. Flow cytometric plots show representative examples of the relative percentage of each population subset from its parent population, while calculated percentages of each population were used to compare the levels of myeloid cells between study cohorts as shown in the scatter plots.

## Results

### Myeloid cells are not expanded in peripheral blood of PBC patients, compared to healthy donors

**Fig. 2:**
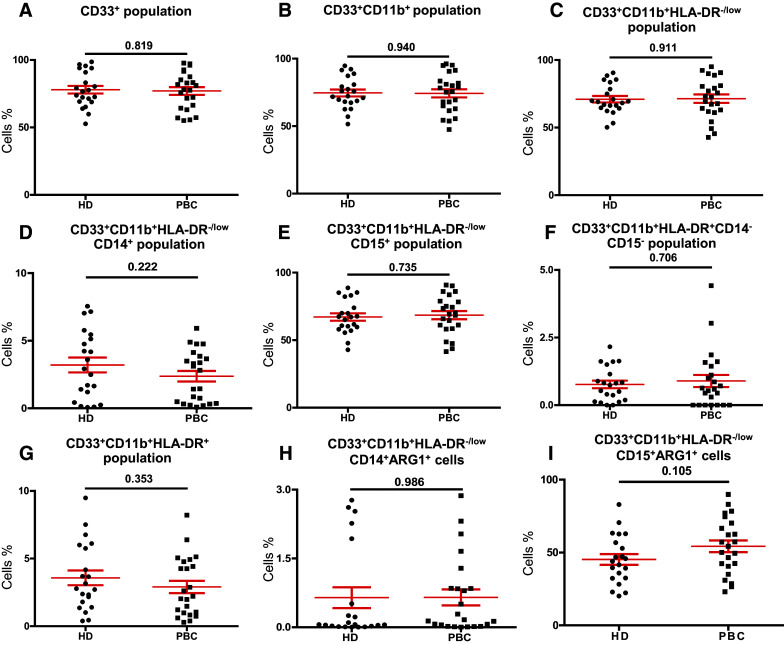
Comparisons of levels of different subsets of circulating myeloid cells between HD and PBC patients. Peripheral blood from 21 HD and 23 PBC patients was stained for myeloid markers. Scatter plots show the mean of calculated percentages ± SEM of CD33^+^ cells (**a**), CD33^+^CD11b^+^ cells (**b**), CD33^+^CD11b^+^HLA-DR^−/low^ cells (**c**), CD33^+^CD11b^+^HLA-DR^−/low^CD14^+^ cells (**d**), CD33^+^CD11b^+^HLA-DR^−/low^CD15^+^ cells (**e**), CD33^+^CD11b^+^HLA-DR^−/low^CD14^−^CD15^−^ cells (**f**), CD33^+^CD11b^+^HLA-DR^+^ cells (**g**), CD33^+^CD11b^+^HLA-DR^−/low^CD14^+^ARG1^+^ cells (**h**) and CD33^+^CD11b^+^HLA-DR^−/low^CD15^+^ARG1^+^ cells (**i**)

In this study, we investigated levels and phenotype of circulating- and tumor-infiltrating myeloid cells. Representative flow cytometric plots for the gating strategy is shown in Fig. 1. Previous studies described human MDSC as cells lacking the expression of markers for mature lymphocytes, monocytes, NK cells and granulocytes [11]. MDSC can be identified as CD33^+^CD11b^+^HLA-DR^−/low^ cells and further categorized into monocytic, granulocytic or immature cells based on the expression or lack of expression of CD14 and CD15. There was no significant increase in the levels of circulating CD33^+^ cells between breast cancer patients and HD (HD; 78.0 ± 2.9 vs PBC; 77.0 ± 2.9, Fig. 2a). Further analysis did not show any expansion in the levels of CD33^+^CD11b^+^ cells (HD; 74.6 ± 2.6 vs PBC; 74.3 ± 3.0; Fig. 2b) and CD33^+^CD11b^+^HLA-DR^−/low^ cells in PBC patients compared with HD (HD; 70.9 ± 2.4 vs PBC; 71.4 ± 3.4; Fig. 2c). Furthermore, PBC patients did not show any expansion in the levels of CD33^+^CD11b^+^HLA-DR^−/low^CD14^+^ M-MDSC (HD; 3.2 ± 0.5 vs PBC; 2.4 ± 0.4, Fig. 2d). Similarly, there was no difference in the levels of circulating CD15^+^ cells within the CD33^+^CD11b^+^HLA-DR^−/low^ populations (HD; 67.1 ± 2.1 vs PBC; 68.5 ± 3.0, Fig. 2e). Indeed, CD33^+^CD11b^+^HLA-DR^−/low^CD15^+^ population includes both neutrophils and G-MDSC due to the phenotypical and functional overlap between the two populations [12]. Thus, we referred to these cells as N/G-MDSC. Additionally, there was no significant expansion in levels of circulating IM-MDSC which lacked expression of both CD14 and CD15 (HD; 0.8 ± 0.1 vs PBC; 0.9 ± 0.2; Fig. 2f).

Cells expressing MHC class II molecule HLA-DR have antigen presenting properties. We compared the levels of CD33^+^CD11b^+^HLA-DR^+^ myeloid cells between cancer patients and HD. Although there was a reduction in levels of circulating antigen-presenting cells (APC) in cancer patients (HD; 3.6 ± 0.5 vs PBC; 2.9 ± 0.5; Fig. 2g), the data did not reach statistical significance.

We also compared the levels of circulating myeloid cells between breast cancer patients (*n* = 23) and female healthy donors (HD(F); *n* = 12). As shown in Supplementary Fig. 1, HD(F) had similar levels of circulating CD33^+^ (HD(F); 79.6 ± 3.7 vs PBC;77.0 ± 2.9), CD33^+^CD11b^+^ (HD(F); 76.9 ± 3.5 vs PBC;74.3 ± 3.0) and CD33^+^CD11b^+^HLA-DR^−/low^ cells (HD(F); 73.3 ± 3.4 vs PBC;71.4 ± 3.1), compared to PBC patients (Supplementary Figs. 1A–C). Likewise, there was no expansion in circulating M-MDSC and N/G-MDSC in PBC patients compared to HD(F) (Supplementary Fig. 1D&E) or in circulating IM-MDSC (HD(F); 0.9 ± 0.2 vs PBC;0.9 ± 0.2) and APC (HD(F);3.5 ± 0.6 vs PBC; 2.9 ± 0.5, Supplementary Figs. 1F&G).

Additionally, we compared the levels of circulating myeloid cells between male and female control donors to rule out any gender dependent effect; there were no significant differences between circulating M-MDSC, G-MDSC, IM-MDSC or APC between the two cohorts (Supplementary Fig. 2). Furthermore, it should be noted that the median age of our HD cohort was lower than that for PBC patients (HD; 29 years vs PBC; 48 years, Table 1).

### ARG1 expression in MDSC subsets

MDSC express high levels of ARG1, which assists in their inhibition of T-cell proliferation and cytotoxicity, expansion of Treg and inhibition of NK cells [13]. We confirmed the immunosuppressive potential of myeloid cells by examining the expression of ARG1. There was no significant difference in the overall levels of ARG1 expressing circulating M-MDSC (HD; 0.6 ± 0.2 vs PBC; 0.7 ± 0.2; Fig. 2h) and N/G-MDSC (HD; 45.3 ± 3.7 vs PBC; 54.3 ± 4.0; Fig. 2i) in breast cancer patients and HD. Furthermore, in line with previous reports [14], our study showed that granulocytic myeloid cells expressed higher levels of ARG1 than monocytic cells in both study groups. Moreover, we found similar results when we compared the levels of circulating myeloid cell subsets with ARG1 expression in HD(F) with PBC patients as shown in Supplementary Fig. 1H&I and also when we compared HD(M) with HD(F) (Supplementary Fig. 2H&I).

### Breast cancer patients with high-stage tumors and/or poorly differentiated tumor cells have similar levels of circulating myeloid cells as those with low-stage tumors and/or well-differentiated tumors

**Fig. 3:**
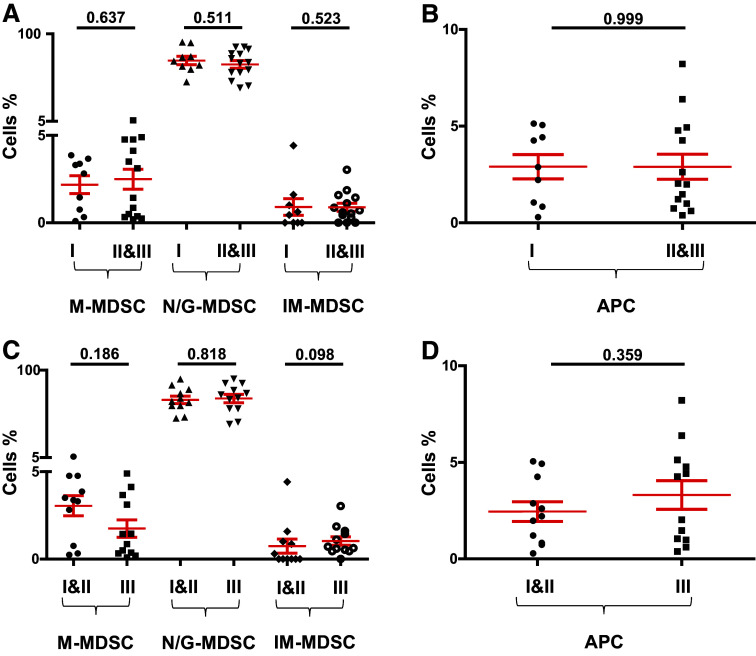
Comparisons of levels of different subsets of circulating myeloid cells between PBC patients with different tumor stages and histological grades. Scatter plots showing calculated percentages ± SEM of CD33^+^CD11b^+^HLA-DR^−/low^CD14^+^ M-MDSC, CD33^+^CD11b^+^HLA-DR^−/low^CD15^+^ N/G-MDSC and CD33^+^CD11b^+^HLA-DR^−/low^CD14^−^CD15^−^ IM-MDSC in patients with stage I (*n* = 9) compared to stage II and III patients (*n* = 14) (**a**) and calculated percentages ± SEM of CD33^+^CD11b^+^HLA-DR^+^ APC (**b**). Scatter plots showing calculated percentages ± SEM of M-MDSC, N/G-MDSC and IM-MDSC in patients with histological grade I and II (*n* = 11) compared to grade III patients (*n* = 12) (**c**) and calculated percentages ± SEM of APC (**d**)

We investigated the potential correlation between levels of circulating myeloid cells with tumor stage and histological grade. Cancer patients were divided into groups based on tumor stage and histological grade, and we compared the levels of circulating myeloid cells between these groups. For TNM stage, we compared patients with stage I tumors (*n* = 9) with those presented with higher tumor stage and regional lymph node invasive stage II and III tumors (*n* = 14). When we compared the levels of circulating myeloid cells between HD and PBC patients with different tumor stages, there were no significant differences between them (data not shown). There were also no differences in the levels of M-MDSC, N/G-MDSC, IM-MDSC and APC between patients with stage I tumors compared with stage II and III patients as shown in Fig. 3a, b. Patients were also divided into two groups based on histological grade: those with well to moderately defined tumor cells with histological grades I and II (*n* = 11), and patients who presented with poorly defined or undifferentiated tumors with histological grade III (*n* = 12). There were no significant differences in the levels of myeloid cells (Fig. 3c) or HLA-DR^+^ APC (Fig. 3d) between patients with different histological grades. Additionally, there was no significant difference in levels of circulating myeloid cells between breast cancer patients with low Ki-67 expression (≤ 30%, *n* = 10) and patients with high Ki-67 expression (>30%, *n* = 8) (*P* values >0.05, data not shown). Similarly, there were no significant differences in levels of myeloid cells between patients positive for estrogen receptor (ER+; *n* = 16) or progesterone receptor (PR+; *n* = 13) compared to ER− (*n* = 7) and PR− (*n* = 10) patients (*P* values >0.05, data not shown).

### Myeloid cells are expanded in breast tumor tissue compared to paired, adjacent non-tumor breast tissue

**Fig. 4:**
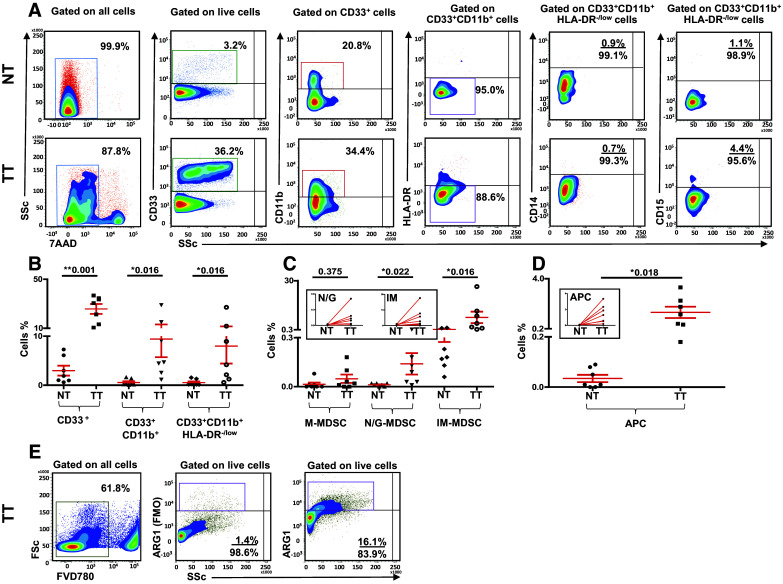
Tissue-infiltrating immune cells. **a** Representative flow cytometric plots showing levels of different subsets of myeloid cells in normal tissue (NT) and corresponding tumor tissue (TT) of 7 PBC patients. Doublets were excluded and live cells were first gated. Scatter plots showing mean of calculated percentages ± SEM of myeloid cell subsets (**b**), and calculated percentages ± SEM of CD33^+^CD11b^+^HLA-DR^−/low^CD14^+^ M-MDSC, CD33^+^CD11b^+^HLA-DR^−/low^CD15^+^ N/G-MDSC and CD33^+^CD11b^+^HLA-DR^−/low^CD14^−^CD15^−^ IM-MDSC in NT and TT of PBC patients (**c**); inset showing the expansion of N/G-MDSC and IM-MDSC in TT compared with adjacent NT. Means of calculated percentage ± SEM of CD33^+^CD11b^+^HLA-DR^+^ APC in NT and TT with inset showing APC expansion in TT compared with adjacent NT (**d**). Flow cytometric plots showing ARG1 expression in cells isolated from TT of one breast cancer patient (**e**). Live cells were gated first using FVD780 and FMO control for ARG1 is shown

One of the main objectives of our study was to investigate phenotype and levels of myeloid cells in the TME of breast cancer patients, compared with paired, adjacent non-tumor normal breast tissue. Representative flow cytometric plots showing differences in levels of myeloid cells between normal tissue (NT) and tumor tissue (TT) of a PBC patient are shown in Fig. 4a. Interestingly, in contrast to peripheral blood, we found significant differences in the levels of myeloid cells in the TME milieu compared with normal breast tissue of seven breast cancer patients (Fig. 4b). The levels of CD33^+^ cells (NT; 3.0 ± 1.0 vs TT; 25.7 ± 4.0), CD33^+^CD11b^+^ cells (NT; 0.6 ± 0.2 vs TT; 9.4 ± 3.7) and CD33^+^CD11b^+^HLA-DR^−/low^ cells (NT; 0.5 ± 0.2 vs TT; 8.0 ± 3.5) were significantly higher in TT compared to NT.

### Expanded myeloid cells in the TME of breast cancer patients are mainly N/G-MDSC and IM-MDSC

To find out if the expanded myeloid subpopulations in the TME of PBC patients are monocytic, granulocytic or immature, we analyzed CD14 and CD15 expression within the CD33^+^CD11b^+^HLA-DR^−/low^ populations (Fig. 4a, c). The levels of CD15^+^ cells were significantly higher in the TME compared with paired, adjacent non-tumor normal breast tissue (NT; 0.01 ± 0.0 vs TT 0.1 ± 0.1). Furthermore, we found a significant increase in the levels of CD33^+^CD11b^+^HLA-DR^−/low^CD14^−^CD15^−^ cells identified as IM-MDSC in tumor tissue (NT; 0.5 ± 0.2 vs TT; 7.8 ± 3.5). In contrast, there was no significant increase in CD14^+^ M-MDSC in tumor compared to normal tissue (NT; 0.01 ± 0.01 vs TT; 0.05 ± 0.02). Interestingly, we also found significantly higher levels of CD33^+^CD11b^+^HLA-DR^+^ cells identified as APC of myeloid origin in the TME (NT; 0.03 ± 0.01 vs TT; 1.4 ± 0.4; Fig. 4d). Importantly, ARG1 was highly expressed in cells isolated from TT (Fig. 4e). However, we could not generate sufficient data for statistical analyses due to limited cell numbers.

## Discussion

Studying the immune profile of the TME in solid cancers is an active area of research. Tumor-infiltrating lymphocytes (TIL), tumor-associated macrophages (TAM), Treg and MDSC are widely recognized as prognostic or predictive markers for cancer progression. Early studies on lymphocytic infiltration in the TME of breast cancer patients showed a close correlation with disease prognosis [15] and were found to have beneficial effects on survival [16]. Several studies have since established certain subsets of TIL, mainly CD8^+^ T cells as a good prognostic factor in various human cancers [3, 17]. In contrast, tumor infiltration and expansion in periphery of immunosuppressive cells has been shown to correlate with poor prognosis and tumor progression in various human malignancies [18]. MDSC and Treg are recognized as key players in the negative regulation of immune responses. Expansion of MDSC has been reported in different human cancers including head and neck, colon, renal, prostate and melanomas [8]. Almand et al. reported an expansion of immature myeloid cells in peripheral blood from patients with head and neck and colon cancers that decreased after removal of tumors from these patients [19]. However, limited data are available on MDSC levels in circulation and matched tumor tissues from cancer patients and their relation with clinical settings. The heterogeneous nature of MDSC due to the varying stages of differentiation at which they were halted, makes it challenging to identify these cells. Furthermore, this gives rise to a morphological mixture of cells, which share many phenotypical and functional characteristics of other cellular populations of myeloid origin. In the present study, we investigated phenotype and levels of tumor-infiltrating and circulating myeloid cells in untreated patients with primary breast cancer.

Several studies have emphasized on the significance of sample handling and processing when monitoring MDSC levels in circulation and are in agreement over the adverse effects of cryopreservation and the delayed time points at which MDSC analysis was carried out following blood collection [20–22]. Mandruzzato et al. suggested to perform analysis on fresh blood to prevent possible loss of some MDSC subsets mainly G-MDSC. Fresh blood analysis also minimizes attenuation of cell surface markers due to Ficoll grade separation [23]. Therefore, we used fresh whole blood for all our analysis on levels of myeloid cells in circulation.

Previous work on MDSC in breast cancer has mainly been performed on murine models. MDSC in mice express CD11b and Gr-1 with monocytic or granulocytic subsets identified by expression of Ly6C and Ly6G, respectively [5]. Accumulating evidence in murine models has shown the significance of MDSC in development and progression of breast cancer [24]. However, these models do not fully reflect the expression levels, cellular functionality of proteins or genes found in human breast tissue [6].

We have recently shown that N/G-MDSC are expanded in the peripheral blood and the TME of patients with colorectal cancer [25]. However, we did not find similar expansion of myeloid cells in circulation of breast cancer patients. Previous studies on MDSC in breast cancer patients have reported an expansion in patients mainly with advanced metastatic disease [26–28]. However, the lack of consistency of markers and criterion to identify MDSC in these studies and more importantly the clinical presentation of study populations might account for discrepancies in results. Diaz-Montero et al. [26] and Solito et al. [27] identified MDSC as Lin^−/low^HLA-DR^−^CD33^+^CD11b^+^ cells in patients with advanced breast cancers, while Yu et al. identified MDSC as CD45^+^CD13^+^CD14^−^CD15^−^ cells with suppressive activity, assessed through IDO expression and reported expansion that correlated with lymph node metastasis in breast cancer patients [28]. Interestingly, they reported that IDO expression was significantly upregulated in tumor-infiltrating MDSC than in periphery, thereby suggesting immunosuppressive role of MDSC in tissue and not in circulation [28]. Additionally, Bergenfelz et al. reported an expansion of CD14^+^HLA-DR^−/low^ monocytic MDSC in circulation in patients with metastatic breast cancer and this expansion correlated with disease severity [29]. Another study showed an expansion of CD33^+^HLA-DR^−/low^CD15^+^CD11b^+^ MDSC in peripheral blood of breast cancer patients with high psychological stress compared to those with lower stress levels, thereby suggesting an association between stress and immune function in breast cancer patients [30].

The main finding in this study is that myeloid cells in the peripheral blood of this breast cancer cohort do not differ when compared to healthy individuals, but when assessing the levels in tumor vs surrounding healthy tissue, the levels were significantly higher in the TME. We found a significant expansion of CD33^+^CD11b^+^HLA-DR^−/low^ myeloid cells in the TME of breast cancer patients. These cells were mainly granulocytic, which include neutrophils and G-MDSC, along with IM-MDSC. Expansion of neutrophils results in suppression of cytolytic activity of immune cells and high NLR is associated with poor prognosis and reduced overall survival in various human malignancies [31]. We also report an expansion of APC of myeloid origin in the TME of PBC patients, based on expression of HLA-DR, which is expressed only on professional APC. APC of myeloid lineage include DC and monocytes. Infiltration of DC has been reported in various solid tumors and has shown to be associated with both good and worse prognosis [32, 33]. The association of tumor-infiltrating DC with worse prognosis in some cancers can be attributed to reduced antigen presentation of DC [34]. Studies have shown tumor-induced functional deficiency of DC in breast cancer patients and reduced antigen-presenting functions in expanded Lin^−^HLA-DR^+^ cells in peripheral blood of cancer patients [35]. Sathhaporn et al. showed defective DC function in peripheral blood from breast cancer patients with decreased IL-12 production, which could assist in tumor progression [36], and Kitchler-Lakomy et al. showed reduced functional activity of DC in peripheral blood of breast cancer patients, which also exhibited immature morphology [37]. Therefore, the expanded HLA-DR^+^ cells in TME in this study require further functional investigation. Furthermore, association between MDSC levels and administration of therapeutic modalities have also been reported. Diaz-Montero et al. reported a significant increase of G-MDSC in breast cancer patients who received adjuvant chemotherapy [26]. MDSC have been proposed as predictive markers for patients’ survival in various diseases. Bailur et al. demonstrated a negative role of MDSC and Treg in the prognosis of breast cancer patients by investigating the association between MDSC and CD8^+^ cells in older untreated breast cancer patients [38].

Breast cancer staging is widely accepted as a useful tool to estimate disease prognosis. Around 5–12% of breast cancer patients presenting with stage I or II tumors die within 10 years of diagnosis compared to over 60% of stage III and over 90% of stage IV patients [39]. Diaz-Montero et al. reported a significant increase of MDSC in peripheral blood from patients with stage IV breast cancers which correlated with metastatic tumor burden [26]. Solito et al. showed that the levels of immunosuppressive MDSC in stage IV advanced breast cancer patients correlated with circulating tumor cells and patients with higher levels had reduced overall survival compared to patients with lower levels [27]. However, we did not find any correlation between circulating myeloid cells and patient staging, arguably due to the fact that patients in our study cohort, in contrast to these studies, presented with initial stages of cancer; none of the patients in our study group presented with distant metastasis. Histologic grading in breast cancers evaluates tubule formation, nuclear pleomorphism and mitotic count [40]. Therefore, tumor histological grades are considered as stage-independent prognostic coefficients that reflect the metastatic potential of the tumor. We divided our cohort into two groups based on histological grades and compared circulating myeloid cell levels between them but did not find any difference between the groups.

Several proinflammatory molecules secreted by tumor cells are responsible for the recruitment of MDSC in the TME where they inhibit immune responses through various mechanisms, such as depletion of nutrients required for lymphocytes, inducing oxidative stress and activating Treg [28]. ARG1 is involved in metabolism of l-Arginine, required for T cell activation and reduces the expression of T cell receptor CD3ζ chain and impairs T-cell response [41, 42]. In line with previous studies [43], we investigated the immunosuppressive potential of myeloid cells by confirming the expression of ARG1 by circulating myeloid cells.

In conclusion, we showed that myeloid cells are expanded in the TME of breast cancer patients, and these cells comprise of immature and granulocytic myeloid cells. Interestingly, we did not find any expansion of myeloid cells in peripheral blood from breast cancer patients. These findings are of great significance in the development of therapeutic agents to target the mechanisms employed by immunosuppressive cells in providing an immune-permissive environment for the progression of cancer.

### Electronic supplementary material

Below is the link to the electronic supplementary material.

Supplementary material 1 (PDF 148 KB)

## Abbreviations

APC: Antigen-presenting cell(s)
ARG1: Arginase 1
DC: Dendritic cell(s)
e-MDSC: Early-stage myeloid-derived suppressor cell(s)
ED: Enzyme disaggregation
ER: Estrogen receptor
FMO: Fluorescence minus one
G-MDSC: Granulocytic myeloid-derived suppressor cell(s)
HD: Healthy donor(s)
HD(F): Healthy donor female(s)
HD(M): Healthy donor male(s)
IM-MDSC: Immature myeloid-derived suppressor cell(s)
MDSC: Myeloid-derived suppressor cell(s)
M-MDSC: Monocytic myeloid-derived suppressor cell(s)
N/G-MDSC: Neutrophil/granulocytic myeloid-derived suppressor cell(s)
NK: Natural killer
NT: Normal tissue
PBC: Primary breast cancer
PBS: Phosphate buffered saline
PMN-MDSC: Polymorphonuclear myeloid-derived suppressor cell(s)
PR: Progesterone receptor
TAN: Tumor-associated neutrophil(s)
TIL: Tumor-infiltrating lymphocyte(s)
TME: Tumor microenvironment
Treg: Regulatory T cell(s)
TT: Tumor tissue
