# Supplementary material for: Myeloid cells in circulation and tumor microenvironment of breast cancer patients
Source: Cancer Immunol Immunother. 2017 Mar 10;66(6):753–64. doi: 10.1007/s00262-017-1977-z (PMC5445142; doi:10.1007/s00262-017-1977-z)
Supplement: Supplementary file 1 — Supplementary material 1 (PDF 148 KB) [file 262_2017_1977_MOESM1_ESM.pdf]

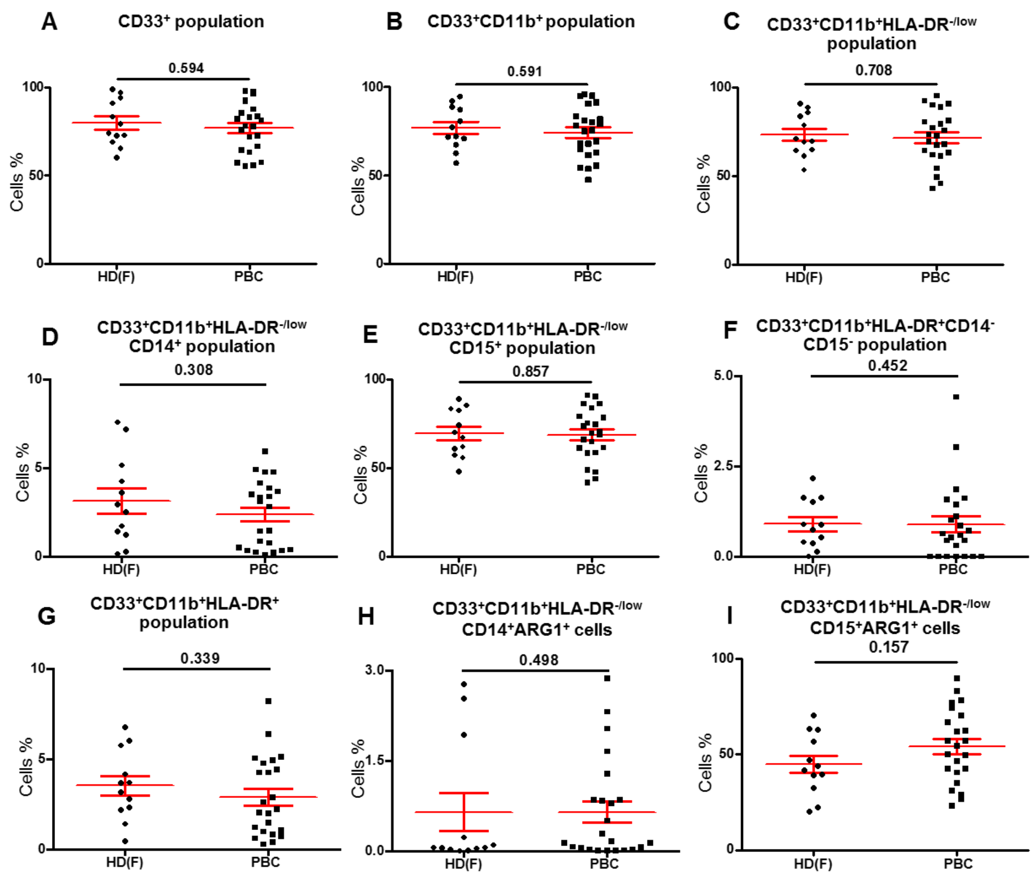


**Supplementary Figure 1: Comparisons of levels of different subsets of circulating myeloid cells between female HD (HD(F)) and PBC patients.** Peripheral blood from 12 female HD and 23 PBC patients was stained for myeloid markers. Scatter plots show the mean of calculated percentages ± SEM of CD33$^{+}$ cells **(A)**, CD33$^{+}$CD11b$^{+}$ cells **(B)**, CD33$^{+}$CD11b$^{+}$HLA-DR$^{-/low}$ cells **(C)**, CD33$^{+}$CD11b$^{+}$HLA-DR$^{-/low}$CD14$^{+}$ cells **(D)**, CD33$^{+}$CD11b$^{+}$HLA-DR$^{-/low}$CD15$^{+}$ cells **(E)**, CD33$^{+}$CD11b$^{+}$HLA-DR$^{-/low}$CD14$^{-}$CD15$^{-}$ cells **(F)**, CD33$^{+}$CD11b$^{+}$HLA-DR$^{+}$ cells **(G)**, CD33$^{+}$CD11b$^{+}$HLA-DR$^{-/low}$CD14$^{+}$ARG1$^{+}$ cells **(H)** and CD33$^{+}$CD11b$^{+}$HLA-DR$^{-/low}$CD15$^{+}$ARG1$^{+}$ cells **(I)**.

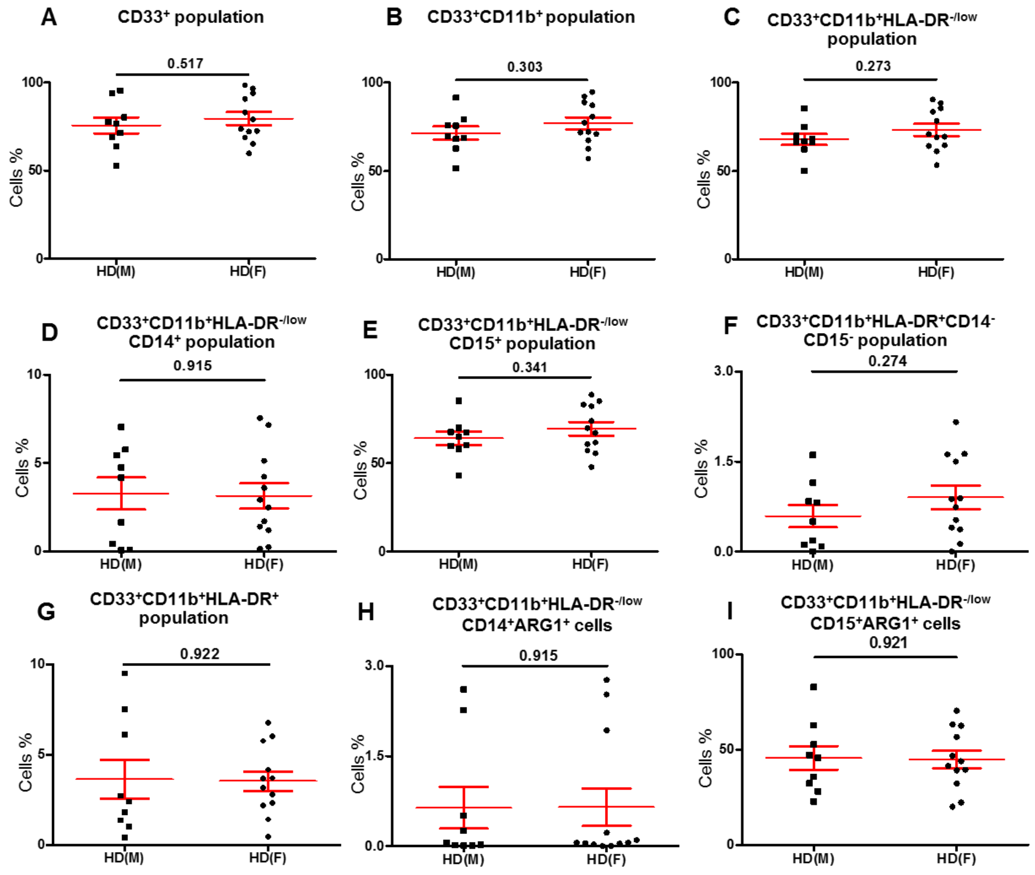


**Supplementary Figure 2: Comparisons of levels of different subsets of circulating myeloid cells between male HD (HD(M)) and female HD (HD(F)).** Peripheral blood from 9 HD(M) and 12 HD(F) was stained for myeloid markers. Scatter plots show the mean of calculated percentages ± SEM of $CD33^+$ cells **(A)**, $CD33^+CD11b^+$ cells **(B)**, $CD33^+CD11b^+HLA\text{-}DR^{-/low}$ cells **(C)**, $CD33^+CD11b^+HLA\text{-}DR^{-/low}CD14^+$ cells **(D)**, $CD33^+CD11b^+HLA\text{-}DR^{-/low}CD15^+$ cells **(E)**, $CD33^+CD11b^+HLA\text{-}DR^{-/low}CD14^-CD15^-$ cells **(F)**, $CD33^+CD11b^+HLA\text{-}DR^+$ cells **(G)**, $CD33^+CD11b^+HLA\text{-}DR^{-/low}CD14^+ARG1^+$ cells **(H)** and $CD33^+CD11b^+HLA\text{-}DR^{-/low}CD15^+ARG1^+$ cells **(I)**.
